# Ordered interpersonal synchronisation in ASD children via robots

**DOI:** 10.1038/s41598-020-74438-6

**Published:** 2020-10-15

**Authors:** Irini Giannopulu, Aude Etournaud, Kazunori Terada, Mari Velonaki, Tomio Watanabe

**Affiliations:** 1 Interdisciplinary Centre for the Artificial Mind (iCAM), FSD, Bond University, 14, University Drive, Robina, Gold Coast, QLD 4229 Australia; 2 Department of Electrical, Electronic and Computer Engineering, Gifu University, Gifu, 501-1193 Japan; 3 Creative Robotics Lab, G Block, University of New South Wales, Greens Rd, Paddington, Sydney, NSW 2021 Australia; 4 Department of Systems Engineering, Prefectural Okayama University, Okayama, 719-1197 Japan

**Keywords:** Human behaviour, Biotechnology, Engineering, Cognitive neuroscience, Development of the nervous system

## Abstract

Children with autistic spectrum disorders (ASD) experience persistent disrupted coordination in interpersonal synchronisation that is thought to be associated with deficits in neural connectivity. Robotic interventions have been explored for use with ASD children worldwide revealing that robots encourage one-to-one social and emotional interactions. However, associations between interpersonal synchronisation and emotional empathy have not yet been directly explored in French and Japanese ASD children when they interact with a human or a robot under analogous experimental conditions. Using the paradigm of actor-perceiver, where the child was the actor and the robot or the human the perceiver, we recorded the autonomic heart rate activation and reported emotional feelings of ASD children in both countries. Japanese and French ASD children showed different interpersonal synchronisation when they interacted with the human perceiver, even though the human was the same in both countries. However, they exhibited similar interpersonal synchronisation when the perceiver was the robot. The findings suggest that the mechanism combining interpersonal synchronisation and emotional empathy might be weakened but not absent in ASD children and that both French and Japanese ASD children do spontaneously and unconsciously discern non verbal actions of non human partners through a direct matching process that occurs via automatic mapping.

## Introduction

Conventionally defined as a disease of neural synchronisation, Autism Spectrum Disorder (ASD) refers to persistent neurodevelopmental deficits in communication and reciprocity vis-à-vis human partners and is associated with restricted and repetitive behavioural patterns whose severity is assessed from level 1 “requiring support” to level 3 “requiring very substantial support”^1^. Studies repeatedly reported that ASD children display a lack of empathy at both emotional and cognitive levels. At the emotional empathy level, ASD children exhibit difficulties synchronising their feelings with others’ feelings; at the cognitive empathy level they demonstrate difficulties considering the others as intentional beings that have thoughts and objectives^2,3^. The aim of the present study was to explore the interpersonal synchronisation and emotional empathy in a perception–action scenario between ASD children and robot versus human in both France and Japan.

A proposed mechanism for the empathetic difficulties associated with ASD emerged from neurophysiological and social studies according to which interactive communication disorders such as, for example, nonverbal (motor) loss may contribute to impairment in empathy^4^. Considering that empathy might lead to “empathetic action”^5^ and investigating the relationship between empathy and behaviour, a flourishing body of research reported interpersonal coordination, that is, reciprocal and direct synchronisation, between companions^6,7^. Similarly, findings from typically developing children supported the triadic relationship between empathy, communication and synchronisation in both human–human and human–robot interaction^8^. Consequently, it seems that interpersonal synchronisation can occur spontaneously^9^ and as such it can be considered a component of idiosyncrasy that concerns both humans and artificial agents, i.e. robots^8,10–12^.

Until now, interpersonal synchronisation perception–action processes have focused on dyadic interactions. In dyadic interactions, one companion naturally accommodates its reactions to the other companion^8,11,12^. Studies revealed that not only typically developing young infants^13^ but also adults^14^ harmonise their face to face, verbal and nonverbal, reactions with their partners. According to the perception–action process, produced synchrony is associated with the experience of the one who initiates the interaction, i.e. the actor, whereas perceived synchrony is the experience of the one who adapts its reactions, i.e. the perceiver^12,15,16^. Even temporarily, it appears that interpersonal synchronisation contributes to and facilitates social and emotional interactions. Intrinsically, interpersonal synchronisation may or may not be employed as an “affiliation tool”, which significantly affects verbal expression and feelings^7,17^. The exaltation of such synchronisation process is possible because of the existence of a dynamic neural network that embraces and embodies cortical (e.g. prefrontal, temporal and parietal cortices) and subcortical (e.g. basal ganglia, hippocampus and cerebellum) areas^18,19^, which essentially lead to construal representations^20^. It is suggested that depicting emotional empathy as an embodied simulation process, which produces and ensures interpersonal synchronisation and attunement, increases connectivity through the sharing of neural cortical and autonomic pathways that soften the barriers between “self” and “other”^10,21–23^. Such a mechanism appears to be overwhelmingly ubiquitous regardless of context and geographic location.

Emotional empathy and synchronisation interdependence appeared to contribute to potential similarities between neurotypical children in France and Japan when the autonomic heart rate activity was scrutinised in both human–human and human–robot interactions^8^. Specifically, using an actor-perceiver scenario, where the actor was a French or a Japanese neurotypical child aged 5 to 6 years old and the perceiver was a human or a robot, several similarities in autonomic heart rate level between the children and within each condition were revealed. In essence, Japanese and French neurotypical children appeared to exhibit similar emotional empathy and synchronisation when they interacted with a human or a robot perceiver. Japanese children displayed similar emotional empathy and synchronisation vis-à-vis the human when the human was the passive or active perceiver, i.e. during the rest condition where no interaction was required, and in the human perceiver condition where the human responded to the child’s speech by nodding only. Additionally, Japanese and French neurotypical children reported similar emotional feelings before and after the interaction with the robot. Interpersonal attunement based on emotional empathy was shown to be correlated with autonomic heart rate modifications not only in typically developing children and infants^24^ but also in adults^25^. Taken together, the aforementioned findings clearly indicate that autonomic functions associated with heart rate do not exclusively depend on genetic factors but are also associated with intercommunication with humans or robots^8^.

Interestingly, ASD children demonstrate disrupted synchronisation in both spontaneous and intentional interpersonal coordination^26,27^. This potential inadequacy in producing interpersonal synchronisation can be thought to depict deficits in neural connectivity^11^ whose apotheosis is reflected in the lack of mirror neuron activity, i.e. broken mirror^28^. Notwithstanding, associations between interpersonal synchronisation and emotional empathy have not yet been directly explored in ASD children when they interact with a human or a robot. Moreover, these associations have never been investigated with ASD children between France and Japan under analogous experimental circumstances.

An important number of studies have shown that children with ASD better interact with animated robots than human partners using various verbal and nonverbal reactions^29–34^. Some of these studies have further demonstrated that ASD children, while engaged with a robot partner, then turn to interact with a third partner, an adult^29,30^. A recent systematic review conducted by Pennisi et al.^35^ confirmed that children with ASD are better encouraged in social and emotional reactions, in general, while they are in contact with animated robots than with humans. Based on predictability and intentionality degrees, reflected in autonomic variations, some other studies indicated that ASD children more efficiently perform empathetic actions with robots than with humans^11,12,36^.

The aforementioned behavioural and neurophysiological reports converge and suggest that emotional empathy is a meaningful pathway for analysing interpersonal synchronisation in ASD children. Essentially, the present study extends our interdisciplinary project on human–human and human–robot interaction^8,11,12^. Associated with the paradigm of speaker-listener, which fundamentally considers the verbal and nonverbal communication between an actor and a perceiver, our studies explored autonomic reactions, i.e. heart rate synchronisation, in neurotypical French and Japanese children^8^. In our paradigm, the actor was always a child (French or Japanese) and the perceiver was a robot or a human. Constructed from a mathematical model and specifically conceived for human–robot interaction and synchronisation, the robot is a free of culture or nation perceiver that repeatedly generates an adapted nodding motion from speech input^37^. In the aforementioned paradigm, the human perceiver was the same person and performed the same experimental procedure in France and Japan. In a recent study conducted with ASD children in France, first we demonstrated that ASD children’s verbal and nonverbal communication is better assisted by a robot’s simplicity and predictability than a human’s complexity and unpredictability^11^. Then, we showed that in neurotypical children aged 5 to 6 years old, emotional empathy as a mechanism of interpersonal synchronisation led to analogous verbal and nonverbal similarities between companions, that is, in human–human and human–robot condition, in both France and Japan^8^. In this study, it is likely that ASD children experienced disrupted interpersonal synchronisation typically based on limitations in emotional empathy, due to reduced neural activity, i.e. neural hypo activation. This brought us to investigate whether these children would synchronise their verbal expressions and autonomic reactions with a human or a robot partner in both France and Japan. Given this, to the best of our knowledge, the present study is one of the first investigations on the autonomic correlates and emotional feeling expressions of ASD children in the context of interpersonal synchronisation and emotional empathy with a human or a robot. As far as autonomic reactions are concerned, since the robot is a free of culture or nation affiliation perceiver specifically designed for human–robot interaction^37^, there is no specific reason to suspect any cultural interferences^8^. We assumed that higher tendencies to synchronise with a robot perceiver would be associated with higher emotional empathy reflected in autonomic reactions (i.e. heart rate and heart rate variability). With that in mind, it was hypothesised that the autonomic reactions of ASD children in France and in Japan would be higher in “with robot” condition than in “with human” condition. If so, it was also hypothesised that in both countries, ASD children would declare feeling better “after” rather than “before” the interaction with the robot partner.

## Methods

### Participants

Two groups of children with ASD, one from France and another from Japan participated in the study. To evaluate the feasibility of the study, an a priori statistical power analysis was conducted using G*Power 3.1^38^ in order to compare the simpler of two matched pairs groups using a one-tailed test, a medium effect size (d = .50), and an alpha of .05. Results showed that a minimum total sample of 27 ASD children was required to achieve a power of .81^39^. However, a total of 40 highly comparable ASD children with two equal sized groups of ASD children in each country (i.e. "French-ASD-Group" and "Japanese ASD-group") was successfully gathered and included in the study. The “French ASD-Group” consisted of twenty children (14 boys and 6 girls), while the “Japanese ASD-Group” was composed of twenty children (17 boys and 3 girls). Male-to-female ratio was approximately 4:1^39^. The groups were comparable in both chronological and developmental age. More particularly, the chronological age of the first group varied from 7 to 9 years old (mean 8 years; sd 3 months), while the developmental age ranged from 6 to 7 years old (mean 6 years; sd 8 months); the chronological age of the second group varied from 7 to 11 years old (mean 9 years; s.d 3 months), and the developmental age ranged from 5 to 7 years old (mean 6 years; s.d 4 months) according to the KBIT 2 Kaufman Brief Test^40^. The mean age when first words appeared was 2 years and 2 months (sd 7 months) for the first group and 3 years and 2 month (sd 3 months) for the second group. Both groups were diagnosed according to the DSM V criteria of ASD^1^ by experienced psychiatrists and psychologists. Both clinical populations consisted of mild-moderate children with ASD as given by the Childhood Autism Rating Scale (CARS)^41^. CARS’ scores were between 31 and 35 for the first group and 32 to 36 for the second group. Both the French and Japanese ASD children were verbal and all attending school classes with specific educational arrangements. The study was approved by both the French (Individual Protection Scientific Committee) and Japanese (Board of Medical Review of the Graduate School of Medicine of Gifu University) local ethics committees and was in accordance with the National ethics committees in France and Japan and the declaration of Helsinki 2.0. In France and Japan, all parents gave their informed consent both verbally and in writing for the participation of their children in the study as well as for the data analysis; however, they did not allow the authors to send out the individual data. Both, in France and Japan, each child was asked to give his/her verbal consent before the study began. Anonymity was guaranteed.

### Materials

#### Robot

**Figure 1:**
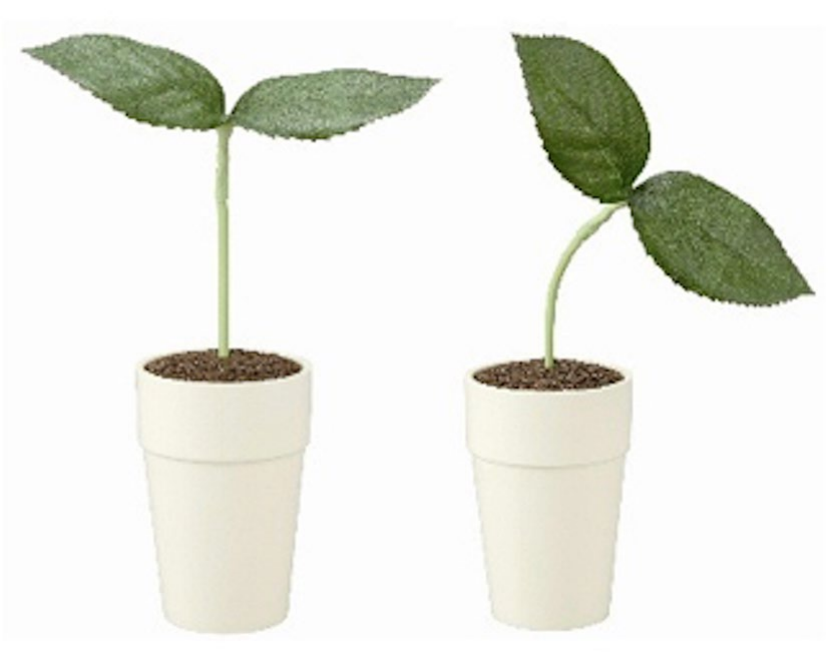
Toy Robot "Pekoppa". The toy robot was used as free of culture or nation affiliation. Pekoppa is the simplest expression of Sakura, which is a voice-driven group of humanoid entrainment systems that reacts to speech sounds by only nodding.

A robot, called “Pekoppa”, was used as the robot perceiver^37^. Pekoppa is the simplest expression of Sakura, which is a voice-driven group of humanoid entrainment systems that reacts to speech sounds, regardless of language, by only nodding. Pekoppa is shaped like a bilobed plant; its leaves and stem make a nodding response based on speech input and supports the sharing of mutual embodiment in communication (Fig. 1). It uses a material called BioMetal made of a shape-memory alloy that acts as its driving force.

#### Heart rate device

The recording of heart rate was possible using a Mio Alpha watch monitor. The watch monitor was placed on the left hand of the participants in both countries. The heart rate was measured online via two green LEDs associated with a photo-electric cell. Both LEDs were incorporated into the back of the watch. Their light penetrates the skin and the photo-electric cell directly detects the volume and the modifications of blood flow. The accuracy of the optical sensor is − 01 ± 0.3 bpm (beats per minute). Note that at the age of 5 to 7 years, the heart rate corresponds to 95 bpm (± 30 bpm).

### Procedure

#### Child and robot conditions

**Figure 2:**
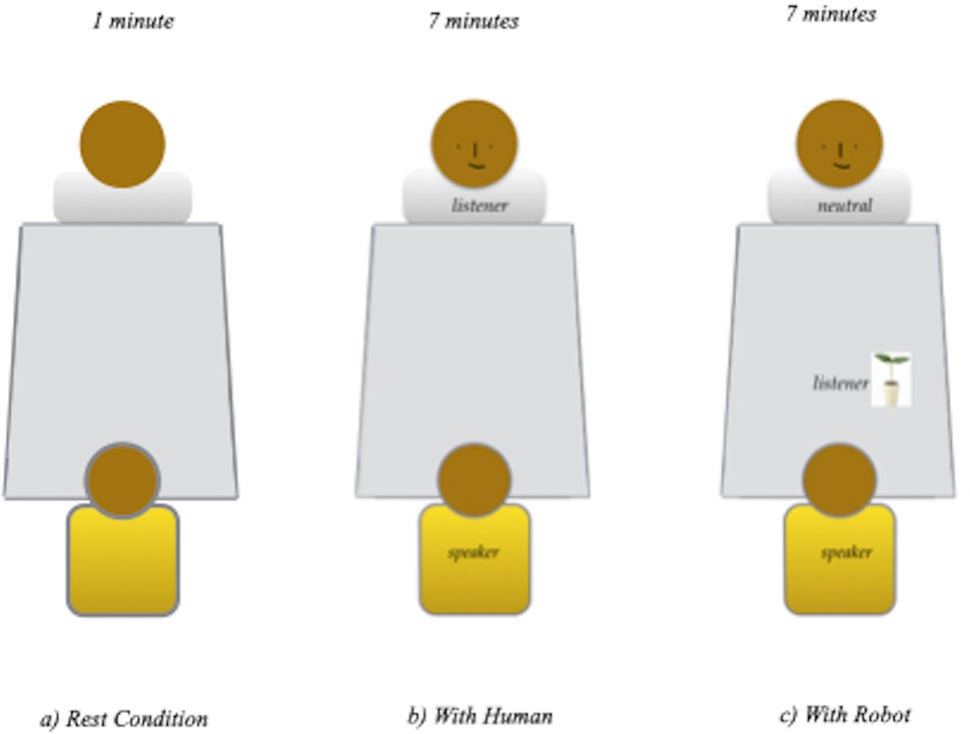
Actor-perceiver paradigm. The actor was always the child; the robot or the human the perceiver.

The paradigm of actor-perceiver^8,10–12^ was used. For both groups, the study took place in a room with which the children were familiar. The room was in schools where participants attended in both France and Japan. Three conditions were defined: the first was called “rest” condition, the second was called “with human” (i.e. ASD child–adult), and the third was called “with robot” (i.e. ASD child–robot). The second and third conditions were counterbalanced across the children. The duration of the “rest" condition was 1 min; the second and third conditions each lasted approximately 7 min. The inter-condition interval was approximately 30 s. For each child, the whole experimental session lasted 15 min (Fig. 2).

At the beginning of each session, the experimenter presented the robot to the child explaining that the robot nods whenever the child speaks. Then, the experimenter hid the robot. The session was run as follows: during the “rest" condition, the heart rate of each child was measured in silence. At the end of that condition, the child was also asked to estimate her/his own emotional feeling on a scale ranging from 1 (the lowest level) to 5 (the highest level)^8,11,12,42,43^. Each level corresponded to a specific emotional state depicted by a child’s face as follows: 1st “Fair”, 2nd “Moderately Good”, 3rd “Good”, 4th “Very Good”, 5th “Excellent”. During the “with human” condition, the child was invited to talk to the experimenter. To that end, the experimenter asked the child “What did you do at school this morning?” As such, the experimenter started a discussion and then listened by only nodding to the child. Meanwhile the heart rate of each child was measured. During the “with robot” condition, the robot was set to nod; the experimenter gave the robot to the child and invited the child to use it. As previously, the experimenter asked the child to tell the robot what he or she did at school this morning. The robot was the perceiver, the child was the actor and the experimenter remained silent and discreet. The heart rate was recorded at the same time once again. The study started at around 10.00 am French and Japanese time for all the children. At the end of each session, the child was invited to estimate his/her own emotion, using the same above-mentioned scale. More particularly, each child was asked to report his/her own emotional feeling after contact with the robot^8,10–12^.

#### Autonomic reaction analysis

The Mio Alpha device was integrated with an iOS device and data were transmitted by Bluetooth technology to an iCardio application. All data consisting of detailed timestamps, were stored and exported for HR (Heart Rate) and HRV (Heart Rate Variability) analysis purposes. The HR (beats per minute) for all French and Japanese ASD children in each condition was considered. Spectral analysis of the HRV was performed based on the two critical frequency domain parameters: low frequency (LF) power (0.04 to 0.15 Hz) and high frequency (HF) power (0.15 to 0.4 Hz). The absolute value of low and high power (ms^2^) was considered to calculate the LF/HF power ratio. The R-R individual intervals time series of each ASD child in each condition was performed via the open-source and compatible MatLab 2019a Toolbox (i.e. HRVTool v1_04). This toolbox has an integrated heart rate detector of R-peak location, but all the R-peaks were also visually inspected to ensure correct identification and classification of every QRS complex. All the collected individual data were then used for statistical analysis.

#### Statistical analysis

Data computation was based on (a) autonomic reaction, i.e. heart rate and heart rate variability (LF/HF ratio) and (b) emotional feeling reported. Data assumption controls were conducted using IBM SPSS Statistics 25 software to determine the appropriateness of the analysis. The assessment of linearity via visual inspection of a scatterplot matrix indicated a violation of this assumption. Mauchly’s test with a Greenhouse–Geisser epsilon (ε) was used to assess sphericity and the resulting value indicated a violation of the assumption. Similarly the Shapiro–Wilk test was applied to assess normality. All data distributions resulted in a level less than .05 signifying a violation of the assumption of normality, which affects Type I and Type II errors resulting in false inferential standards. Based on the above, a Wilcoxon related signed rank test was accomplished to compare heart rate and heart rate variability medians within the French ASD group on the one side and the Japanese ASD group on the other side. A Mann–Whitney U test was carried out to compare heart rate, heart rate variability and emotional feeling medians between the French and Japanese ASD children.

## Results

First, the results for the heart rate and heart rate variability (i.e. LF/HF ratio), in three conditions: “rest”, “with human”, “with robot” at both between and within group levels will be presented. Then, the emotional feeling reported for each ASD children group once again at both between and within levels will be examined.

**Figure 3:**
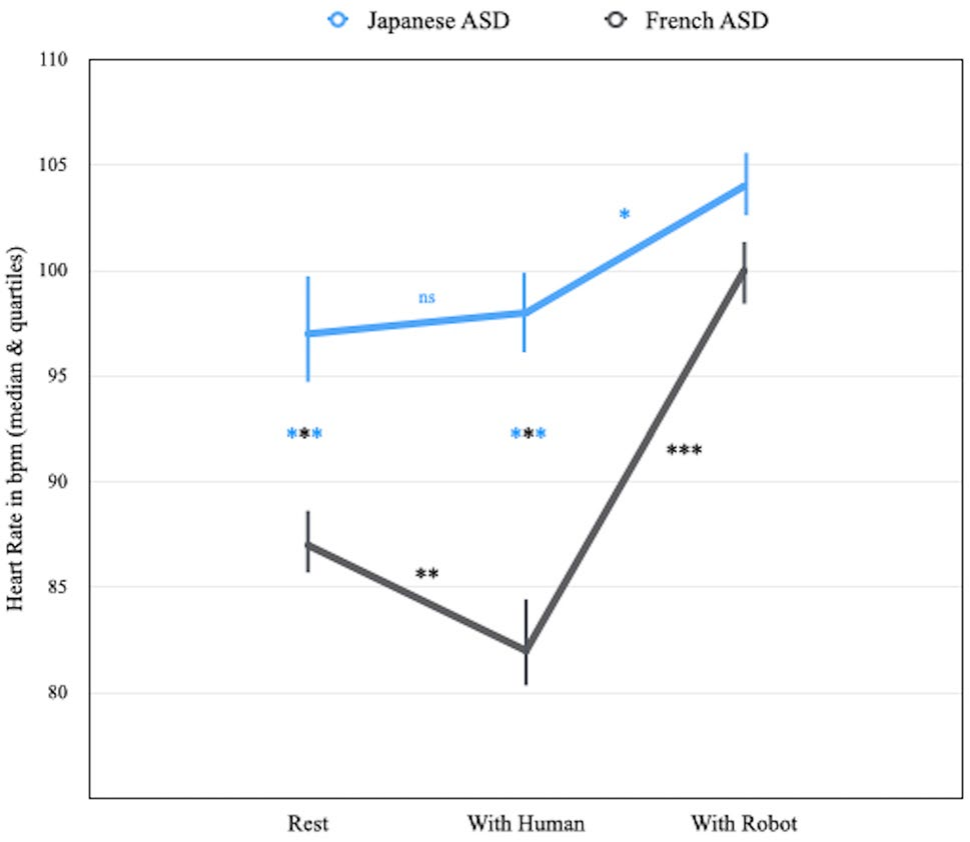
Heart rate comparison between Japanese and French ASD children in ‘rest’, ‘with human’ and ‘with robot’ condition (*p < 0.05; **p < 0.01; *** p < 0.0001). (**a**) Japanese ASD-group showed higher heart rate than French children when the partner was the human. (**b**) Heart rate of both French and Japanese ASD-groups were very similar when the partner was the robot.

When between-group comparisons were analysed (Fig. 3), the median heart rate of the Japanese ASD children was higher (Median = 97) than the median heart rate of the French children (Median = 87) in the “rest condition” (Mann–Whitney U = 85.5, *p* < 0.001). Analogously, the median heart rate of the Japanese ASD children (Median = 98) was higher than the median heart rate of the French ASD children (Median = 82) when the perceiver was the human (Mann–Whitney U = 20.50, *p* < 0.001). However, the median heart rate of the Japanese ASD children (Median = 104) did not differ from the median heart rate of the French ASD children (Median = 100) when the perceiver was the robot (Mann–Whitney U = 199.5, *p* = non-sig).

When within-group comparisons were examined, the median heart rate in the "rest" (Median = 97) condition was similar to the median heart rate in the “with human” (Median = 98) condition for the Japanese ASD children (Wilcoxon T = 116, *p* > 0.05), but it was lower in the "with human” (Median = 82) condition than in the “rest" (Median = 87) condition for the French ASD children (Wilcoxon T = 24, *p* < 0.01). The median heart rate in the “with robot” condition was higher (Median = 104) than the median heart rate (Median = 97) in the “rest"condition for the Japanese ASD children (Wilcoxon T = 66, *p* < 0.05 for the Japanese). Similarly the median heart rate in the "with robot" condition was higher (Median = 100) than the median heart rate (Median = 87) in the “rest" condition for the French ASD children (Wilcoxon T = 210, *p* < 0.001). The median heart rate of the Japanese ASD children was higher when they interacted with the robot perceiver (i.e. “with robot” condition, Median = 104) than when they interacted with the human perceiver (i.e. “with human” condition, Median = 98) (Wilcoxon T = 64, *p* < 0.05). In addition, the median heart rate of French ASD children was higher in the "with robot" condition (Median = 100) than in the "with human" (Median = 82) condition (Wilcoxon T = 1, *p* < 0.001).

**Figure 4:**
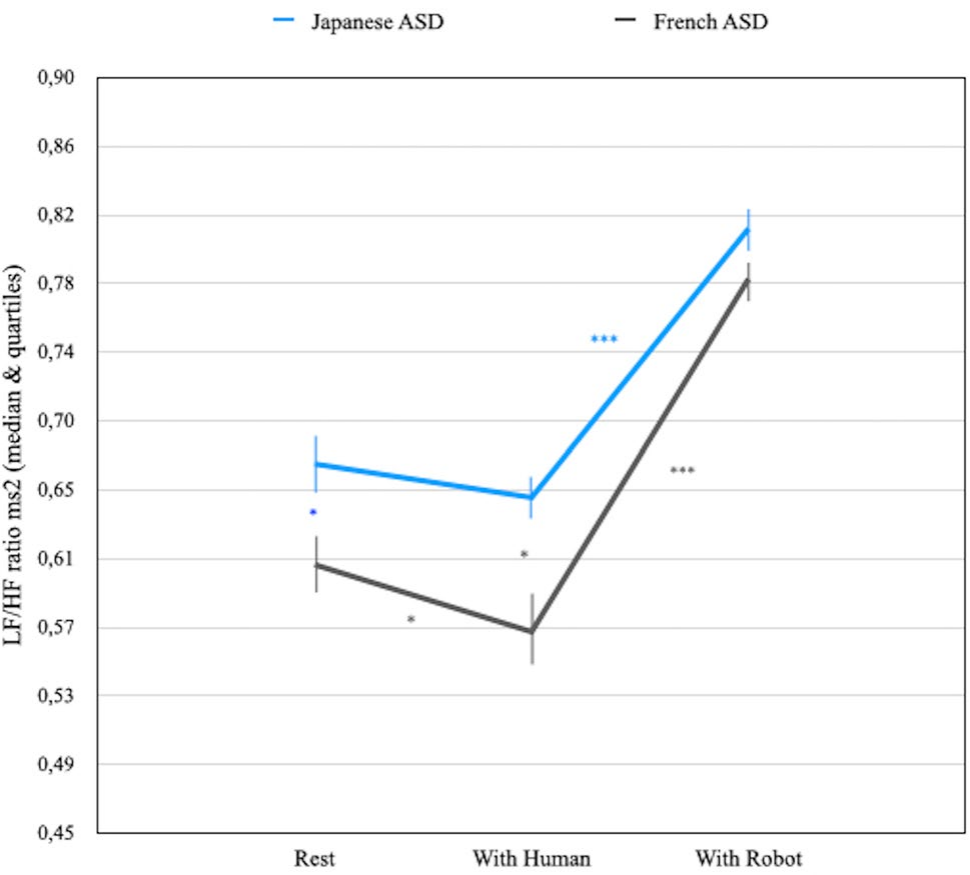
Heart rate variability (i.e. LF/HF ratio) comparison between Japanese and French ASD children in ‘rest’, ‘with human’ and ‘with robot" condition (*p < 0.05; *** p < 0.0001). (**a**) Japanese ASD-group demonstrated higher LF/HF ratio than French children when the partner was the human. (**b**) LF/HF ratio of both French and Japanese ASD-groups were very similar when the partner was the robot.

Figure 4 illustrates the between-group comparisons with regard to heart rate variability, based on the LF/HF ratio. A Mann–Whitney U test indicated that the median LF/HF ratio was higher for Japanese ASD children (Median = 0.67) than for French ASD children (Median = 0.61) in the "rest" condition (Mann–Whitney U = 118.5, *p* < 0.05). Interestingly, when the perceiver was the human, the median LF/HF ratio of the Japanese ASD children was higher (Median = 0.65) than the median LF/HF ratio (Median = 0.57) of the French ASD children (Mann–Whitney U = 121, *p* < 0.05). Finally, when the perceiver was the robot, the median LF/HF ratio (Median = 0.81) of the Japanese ASD children did not differ from the median LF/HF ratio (Median = 0.78) of the French ASD children (Mann–Whitney U = 137, *p* = non-sig).

With respect to the within-group comparisons, a Wilcoxon related signed rank test showed that the median LF/HF ratio in "rest" (Median = 0.67) and "human" (Median = 0.65) condition did not differ for the Japanese ASD children (Wilcoxon T = 90, *p* = non-sig). On the contrary, the median LF/HF ratio was higher in the "rest" (Median = 0.61) than in the "with human" (Median = 0.57) condition for the French ASD children (Wilcoxon T = 35.5, *p* < 0.05). A Wilcoxon related signed rank test indicated that the median LF/HF ratio was lower in the "rest" (Median = 0.67) than in the "with robot" (Median = 0.81) condition (Wilcoxon T = 3, *p* < 0.001) for the Japanese ASD children. It also appears that the median LF/HF ratio was lower in the "rest" (Median = 0.61) than in the "with robot" (Median = 0.78) condition for the French ASD children (Wilcoxon T = 0, *p* < 0.001). Lastly, the median LF/HF ratio was analysed in the "with robot" and the "with human" conditions for Japanese on the one hand and French ASD children on the other hand. The median LF/HF ratio in the "with robot" (Median = 0.81) was higher than in the "with human" (Median = 0.65) condition for Japanese ASD children (Wilcoxon T = 171, *p* < 0.001). A similar pattern of statistical results was observed for the French ASD children according to which the median LF/HF ratio was higher in the "with robot" condition (Median = 0.78) than in the "with human" (Median = 0.57) condition (Wilcoxon T = 210, *p* < 0.001).

**Figure 5:**
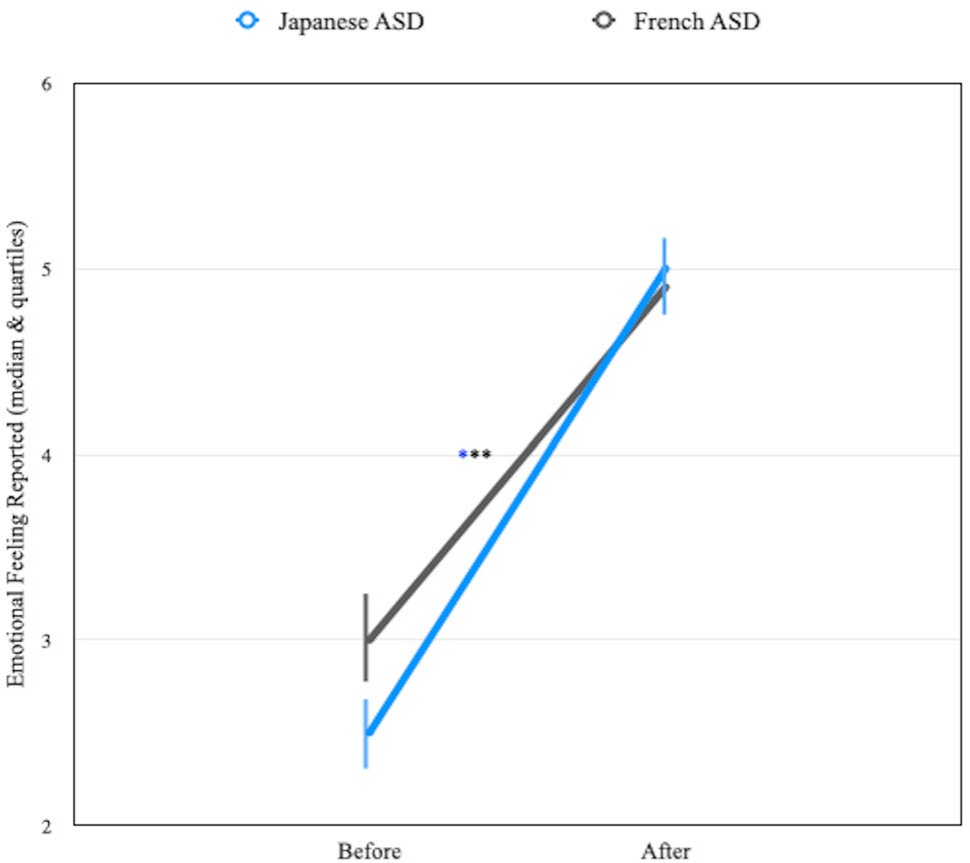
Comparison of Emotional feeling reported ‘before’ and ‘after’ the interaction with the robot in Japanese and French ASD children (***p < 0.001). (**a**) Very similar emotional feelings for both French and Japanese ASD children “before” and "after" the interaction with the robot. (**b**) Both groups significantly increased their feelings when the partner was the robot and declared that they felt better “after” than “before” the interaction with the robot.

Figure 5 represents the feelings expressed by the children "before" and "after" the interaction with the robot. The between-group comparisons show that the emotional feelings reported by the ASD children were very similar "before" (Median = 2.5 for the Japanese ASD children and Median = 3 for the French ASD children; Mann–Whitney U = 158, *p* = non-sig) and "after" the interaction with the robot (Median = 5 for the Japanese ASD children and Median = 4.9 for the French ASD children; Mann–Whitney U = 213, *p* = non-sig). The within-group comparisons also show that the emotional feeling of the Japanese ASD children was better "after" (Median = 5) than "before" (Median = 2.5) the interaction with the robot (Wilcoxon T = 98, *p* < 0.01). Similarly, the emotional feeling reported by the French ASD children was better "after" (Median = 4.9) than "before" (Median = 3) the interaction with the robot (Wilcoxon T = 210, *p* < 0.001).

## Discussion

In the present study, we sought to experimentally analyse interpersonal synchronisation between partners, i.e. ASD children and robot versus human in France and Japan. To that end, children were put in a perception–action scenario represented by the speaker-listener paradigm where the children always took the role of the actor while the perceiver was the robot or the human. The robot was built to follow the actor’s behaviour in a synchronous way, i.e. nodding; the human was instructed to react in the same way. Autonomic reactions reported by the heart rate, heart rate variability, and emotional feelings reported verbally were recorded.

Physiological findings revealed that the Japanese ASD children had higher autonomic reactions than the French children both in the “rest” and in “with human” conditions. Nonetheless, the autonomic reactions of both the French and the Japanese ASD children were very similar in the “with robot” condition. The autonomic reactions of the French ASD children were lower in the “with human” condition than in the “rest” condition and they increased and became higher in the “with robot” condition compared to the two other conditions. The autonomic reactions (i.e. HR and HRV) of the Japanese ASD children in the “rest” condition were similar to the autonomic reactions in the “with human” condition, but they were higher in the “with robot” condition than in the "rest" and “with human” conditions. It appears that the Japanese and French ASD children showed different interpersonal synchronisation when they interacted with the human perceiver, even though the human was the same in both countries. However, they exhibited similar autonomic reactions and thus similar interpersonal synchronisation when the partner was the robot. Behavioural findings reported very similar emotional feelings for both the French and Japanese ASD children “before” and "after" the interaction with the robot. More precisely, both groups of children declared that they felt better “after” than “before” the interaction with the robot.

Considering the above, the present analysis reported differential autonomic activities between the French and Japanese ASD children when the perceiver was the human. Expressly, the ASD Japanese children exhibited higher empathetic reactions than the French ASD children in both "rest" and "with human" conditions. With regard to the "rest" condition, the findings are consistent with the profile of differences in autonomic activities observed between French and Japanese neurotypical children^8^. As French neurotypical children showed lower heart rate than Japanese neurotypical children in the "rest" condition in the above mentioned study, French ASD children had lower autonomic reactions than Japanese ASD children in the current study. Contrary to the empathetic reactions of both French and Japanese neurotypical children who displayed very similar profiles in the "with human" condition, the present findings depict significant different empathetic reactions between ASD children. One could expect that these differences might be explained with reference to the cultural background, given the nature of the study and that the human experimenter did not have any Japanese instructional experience. If this cultural assumption was correct, this would mean that neurotypical French and Japanese children would exhibit significant differences in both "rest" and "with human" conditions. However, this was not the case, as the findings reported above demonstrated^8^. Note that on the one hand, the non human perceiver was a free of culture or nation affiliation robot specifically conceived for human–robot intercommunication and that on the other, the measure used to assess empathetic reactions was an autonomic one: heart rate and heart rate variability (i.e. LF/HF ratio). The observed differences can be understood in the clinical context of the disorder rather than in a cultural one. It is in the nature of the Autism Spectrum Disorder (ASD), because it is extended on a spectrum, to be highly heterogeneous and such heterogeneity still seems to be present even though in the current study, many precautions were taken to homogenise the samples. Note that ASD diagnosis is based on specific symptoms defined by the DSM^1^ manual where no cultural specifications or references are exhibited and considered. Such autonomic behaviour can also be understood as a characteristic reaction of ASD children due to the fact that these children exhibit different autonomic activities according to the degree of predictability or unpredictability^11^ or the degree of intentionality of the perceiver^23^.

The current findings also showed that the ASD children both in France and Japan reported more empathetic reactions (verbal and nonverbal) towards the robot than the human partner. These results are consistent with our hypothesis according to which, the autonomic activities would be more noticeable for the robot than for the human. That is, both the French and Japanese ASD children would be better synchronised with the robot perceiver than the human perceiver. Such results are also in line with previous findings which reported that ASD children display a significantly higher heart rate, when they are in contact with a robot than when they are in contact with a human^11,12^. It is noteworthy that in the robot perceiver situation, the autonomic reactions of both the French and Japanese ASD children increased and became analogous to the autonomic activities exhibited by French and Japanese neurotypical children in the same condition. In other words, the French and Japanese ASD children’s autonomic reactions were different when the human was the perceiver but very similar when the perceiver was the robot. Interestingly, the Japanese and French ASD children’s autonomic heart rate reactions increased vis-à-vis the robot perceiver but never exceeded the normal physiological limits. The data suggest that higher synchronisation during dyadic child-robot interaction would be related with higher emotional empathy, i.e. empathetic actions. This was similar for both Japanese and French children. The findings are not only consistent with several studies demonstrating a positive effect of interpersonal synchronisation, as in cooperation and affiliations^7,15^, but they also enrich this approach by suggesting that both the Japanese and French ASD actor’s synchronisation during interaction with a robot partner increases the actor’s autonomic reactions. Such synchronisation likely reflects the way the ASD actor is able to be with the “other” when the "other" is a non human partner: a robot. Both the French and Japanese ASD children verbally reported such preference. Specifically, contrary to the French and Japanese neurotypical children’s reports, they declared better emotional feelings "after" than "before" the interaction with the robot. Consistent with the hypothesis of the present study, this finding is constantly reported when interpersonal interaction with robots is concerned: synchronisation with robots improves ASD children’s emotional feelings^11,12^. As such, it would appear to be a robust result, and would signify that the robot perceiver not only harmonised the autonomic reactions of the French and Japanese ASD children, but also gave them the possibility to enhance their verbal communicative abilities.

With regard to the ASD-robot interaction, we can speculate on the autonomic mechanism underlying the link between interpersonal synchronisation and emotional empathy. It was demonstrated that interpersonal synchronisation involving autonomic activity (i.e. heart rate) would be directly and indirectly connected to cortical (prefrontal, temporal and cingulate) areas and associated with emotional empathy in neurotypical children both in France and Japan^8^. It was also suggested that autonomic activation of ASD children would significantly increase when the perceiver was a robot instead of a human^11^. The observed increase is assumed to be a state of "mobilisation" that mirrors the emotional (and social) engagement of ASD children towards the robot. Taking into consideration the above, the present results are coherent with the hypothesis that non human partners, i.e. robots, would improve the brain activity of ASD children^43,44^. Interestingly, even though the children displayed different autonomic reactions with the human partner, an analogous "mobilisation" mechanism seemed to be present in both the French and Japanese ASD children only with the robot partner. By increasing the autonomic reactions, the Japanese and French ASD children improved their interpersonal synchronisation with the robot. In other words, they were spontaneously better interconnected with the non human than the human partner. Using temporal dynamic brain activations during dyadic coordinated actions, EEG studies suggest that interbrain oscillatory coupling recruiting prefrontal, temporal and parietal regions might play a significant role in interpersonal nonverbal attunement^45^. This suggestion is coherent with several simulation hypotheses according to which observed actions of others are reflected not only on the behavioural but also on neuronal level and as such, they allow people to share and understand their thoughts and feelings^4,8,22^. In our situation, by increasing autonomic reactions, which in turn would increase the associated neural activity and interpersonal synchronisation, the robot, not the human, served as valuable perceiver for the ASD children in both France and in Japan.

Thus, when analysing the participants’ autonomic reactions, both the Japanese and French ASD children were better synchronised with the robot than with the human. The current findings are not only similar to several data reporting that ASD children interact better with robots than with humans^29,30,32–34,43,44^, but go beyond the existing results as we demonstrated for the first time, to our knowledge, that ASD children are involved in spontaneous interpersonal synchronisation in both France and Japan. These findings can be associated with the human inclination to enact in synchrony with machines or humans without being aware of it^8,12,35^. However, where ASD children are concerned, interpersonal synchronisation reinforces the emotional or social connection experienced between the individual and the robot through an automatic process (i.e. the heart rate and heart rate variability). These observations are in line with the idea that individuals with ASD are capable of interpersonal synchronisation at an automatic level, when the social and emotional load is lower, that is, when they interact with the robot, rather than when they interact with the human. In other words, and once again coherent with previous findings, these results suggest that ASD children, in both France and Japan, might be more reliant on low-level intentionality than on high-level intentionality, namely more on robots than on humans^12^. Such a hypothesis is reinforced by the fact that, even though the human partner was the same performing the same experimental procedure in both countries, the French and Japanese ASD children showed analogous autonomic activities only with the robot partner.

Taken together with the existing data in ASD-robot interaction, the present findings might signify that the tendency to generate spontaneous interpersonal synchronisation with robots would be a common characteristic among moderate ASD children. The findings also suggest that the mechanism combining interpersonal synchronisation and emotional empathy might be weakened but not absent, in ASD children and that both French and Japanese ASD children do spontaneously and unconsciously ”understand” non verbal actions of non human partners through a direct matching process that occurs via automatic mapping. This seems to indicate that non human partners would probably reduce synchronisation deficits in neural connectivity. With that in mind, it can be suggested that the above observations are consistent with adaptive resonance theory explaining how the brain automatically reacts and learns from the interaction with events (i.e. nodding) in a changing environment^46^. Given that an autonomic reaction, i.e. the heart rate and heart rate variability, is considered as reflecting the interpersonal cooperation between partners (i.e. human versus robot) and the reporting of the feelings concerning the emotional verbal autoevaluation of the robot’s cooperation, it raises the question of intentional (i.e. cognitive-conscious) and spontaneous (i.e. automatic-nonconscious) synchronisation.

To our knowledge, this is the first time that both approaches (conscious versus unconscious) have been combined in an ASD-robot interaction based on an identical experimental design performed by the same human perceiver in France and Japan. The robot perceiver was a minimalist interactor robot, the simplest expression of Sakura, which is a humanoid entrainment robotic system for sharing interpersonal communication by nodding only. The robot is a free of culture or nation affiliation perceiver characterised by a minimum of socialness, fundamentally expressed by its nodding responsiveness to the interlocutor’s utterances, regardless of the language^37^. The better synchronisation of heart rate and heart rate variability of Japanese and French ASD children with the robot might be understood as being related to the children’s preferences, and more particularly to their inclination for their attention to be drawn to minimalist objects to which they can designate their own or other mental states^11^. Such an inclination, can be thought of as reflecting the children’s yearning to communicate with humans using the robot perceiver. This speculation is possible because the robot is, in fact, a miniature of a human listener whose head nodding responses (i.e. non verbal communication) appear to be handled by ASD children in both countries in a similar way to which neurotypical children commonly do with humans. Coherent with previous studies^8,10–12^, these findings strongly support the idea that minimalistic robots may be a potential way to scrutinise ASD autonomic and cortical embrainment processing^23^. Even so, it seems conceivable that the transformation of a spontaneous non human partner’s synchronisation into an understanding of the human mental state would necessitate more complex processing and require longer duration, which our minimalistic interpersonal paradigm did not provide. We suggest that interpersonal synchronisation during minimalistic social interaction might be related to empathetic actions in both French and Japanese ASD children and that this mechanism, which is both autonomic and controlled (verbal expression) might be inhibited, but not absent, in ASD children.

Limitations of the current study include the exploratory nature of the experimental analyses in both countries. Among the strengths of the present study is the experimental paradigm that it offers. For instance, rather than awaiting natural variations in synchrony between two human partners, i.e. ASD child and human only, the current study permits the use of robots and compares the interpersonal synchronisation between human–robot and human–human. Finally, the current perception–action scenario is an experimental paradigm that could be used for future studies and analyses between, for instance, neurotypical and neurodiverse ASD children from all levels of severity. Future studies could also investigate the degree of synchronicity and asynchronicity in the human–human and human–robot inter-communication in relationship with various autonomic activities including heart rate, electrodermal activity and respiration.

## References

[1] American Psychiatric Association. Autism spectrum disorders. In *Diagnostic and Statistical Manual of Mental Disorders* 5th edn (2013).

[2] Baron-Cohen, S. Theory of mind and autism: a review. In *International Review of Research in Mental Retardation: Autism*, Vol. 23, 169–184 (Academic Press, 2001).

[3] Dziobek I (2008). Dissociation of cognitive and emotional empathy in adults with Asperger syndrome using the Multifaceted Empathy Test (MET). J. Autism Dev. Disord..

[4] Gallese V (2003). The roots of empathy: the shared manifold hypothesis and the neural basis of intersubjectivity. PSP.

[5] Gerdes KE (2011). Empathy, sympathy, and pity: 21st-century definitions and implications for practice and research. J. Soc. Serv. Res..

[6] Chartrand TL, Lakin JL (2013). The antecedents and consequences of human behavioral mimicry. Annu. Rev. Psychol..

[7] Koehne S, Hatri A, Cacioppo JT, Dziobek I (2016). Perceived interpersonal synchrony increases empathy: insights from autism spectrum disorder. Cognition.

[8] Giannopulu I, Terada K, Watanabe T (2018). Emotional empathy as a mechanism of synchronisation in child–robot interaction. Front. Psychol..

[9] Issartel J, Marin L, Cadopi M (2007). Unintended interpersonal co-ordination: ‘can we march to the beat of our own drum?’. Neurosci. Lett..

[10] Giannopulu I, Nakatsu R (2017). Enrobotment: toy robots in the developing brain. Handbook of Digital Games and Entertainment Technologies.

[11] Giannopulu I, Montreynaud V, Watanabe T (2016). Minimalistic toy robot to analyze a scenery of speaker-listener condition in autism. Cogn. Process..

[12] Giannopulu I, Terada K, Watanabe T (2018). Communication using robots: a perception–action scenario in moderate ASD. J. Exp. Theor. Artif. Intell..

[13] Tramacere A, Pievani T, Ferrari PF (2017). Mirror neurons in the tree of life: mosaic evolution, plasticity and exaptation of sensorimotor matching responses. Biol. Rev..

[14] Cornejo C, Cuadros Z, Morales R, Paredes J (2017). Interpersonal coordination: methods, achievements, and challenges. Front. Psychol..

[15] Cacioppo S (2014). You are in sync with me: neural correlates of interpersonal synchrony with a partner. Neuroscience.

[16] Lischke A (2018). Heart rate variability is associated with psychosocial stress in distinct social domains. J. Psychosom. Res..

[17] Tatsukawa K, Nakano T, Ishiguro H, Yoshikawa Y (2016). Eyeblink synchrony in multimodal human–android interaction. Sci. Rep..

[18] Cangelosi A (2010). Grounding language in action and perception: from cognitive agents to humanoid robots. Phys. Life Rev..

[19] Fedorenko E, Hsieh P-J, Nieto-Castañón A, Whitfield-Gabrieli S, Kanwisher N (2010). New method for fMRI investigations of language: defining ROIs functionally in individual subjects. J. Neurophysiol..

[20] Marshal D, Quinn P, Lea SEG (2010). The Making of Human Concepts.

[21] Lamm C, Decety J, Singer T (2011). Meta-analytic evidence for common and distinct neural networks associated with directly experienced pain and empathy for pain. Neuroimage.

[22] Gallese V, Sinigaglia C (2011). What is so special about embodied simulation?. Trends Cogn. Sci. (Regul. Ed.).

[23] Giannopulu I (2018). Neuroscience, Robotics and Virtual Reality: Internalised vs Externalised Mind/Brain.

[24] Feldman R, Magori-Cohen R, Galili G, Singer M, Louzoun Y (2011). Mother and infant coordinate heart rhythms through episodes of interaction synchrony. Infant Behav. Dev..

[25] Stellar JE, Cohen A, Oveis C, Keltner D (2015). Affective and physiological responses to the suffering of others: compassion and vagal activity. J. Pers. Soc. Psychol..

[26] Dinstein I (2011). Disrupted neural synchronization in toddlers with autism. Neuron.

[27] Fitzpatrick P (2016). Impairments of social motor synchrony evident in autism spectrum disorder. Front. Psychol..

[28] Ramachandran VS, Oberman LM (2006). Broken mirrors: a theory of autism. Sci. Am..

[29] Kozima H, Michalowski MP, Nakagawa C (2009). Keepon. Int. J. Soc. Robot..

[30] Giannopulu I, Pradel G (2010). Multimodal interactions in free game play of children with autism and a mobile toy robot. NeuroRehabilitation.

[31] Diehl JJ, Schmitt LM, Villano M, Crowell CR (2012). the clinical use of robots for individuals with autism spectrum disorders: a critical review. Res. Autism Spectr. Disord..

[32] Srinivasan SM (2015). The effects of rhythm and robotic interventions on the imitation/praxis, interpersonal synchrony, and motor performance of children with autism spectrum disorder (ASD): a pilot randomized controlled trial. Autism Res. Treat..

[33] Srinivasan SM, Eigsti I-M, Neelly L, Bhat AN (2016). The effects of embodied rhythm and robotic interventions on the spontaneous and responsive social attention patterns of children with autism spectrum disorder (ASD): a pilot randomized controlled trial. Res. Autism Spectr. Disord..

[34] Kumazaki H (2018). Can robotic systems promote self-disclosure in adolescents with autism spectrum disorder? A pilot study. Front. Psychiatry.

[35] Pennisi P (2016). Autism and social robotics: a systematic review. Autism Res..

[36] Pierno AC, Mari M, Lusher D, Castiello U (2008). Robotic movement elicits visuomotor priming in children with autism. Neuropsychologia.

[37] Watanabe T, Fukuda S (2011). Human-entrained embodied interaction and communication technology. Emotional Engineering: Service Development.

[38] Faul F, Erdfelder E, Lang AG, Buchner A (2007). G*Power 3: a flexible statistical power analysis program for the social, behavioral, and biomedical sciences. Behav. Res. Methods.

[39] Irimia A, Torgerson CM, Jacokes ZJ, Van Horn JD (2017). The connectomes of males and females with autism spectrum disorder have significantly different white matter connectivity densities. Sci. Rep..

[40] Kaufman, A. S. & Kaufman, N. L. KABC-II - Batterie pour l’examen psychologique de l’enfant - 2ème édition - Pearson Clinical & Talent Assessment (2008).

[41] Schopler E, Reichler RJ, De Vellis RF, Daly K (1980). Toward objective classification of childhood autism: Childhood Autism Rating Scale (CARS). J. Autism Dev. Disord..

[42] Giannopulu I, Sagot I (2010). Ressenti émotionnel positif dans une tâche expérimentale chez l’enfant. Ann. Méd. Psychol. Revue Psychiatr..

[43] Giannopulu, I. Cognitive and emotional interactions between autistic child, mobile robot and therapist: a case report. 10.3389/conf.fncom.2011.52.00002/event_abstract.

[44] Giannopulu I (2013). Multimodal interactions in typically and atypically developing children: natural versus artificial environments. Cogn. Process..

[45] Yun K, Watanabe K, Shimojo S (2012). Interpersonal body and neural synchronization as a marker of implicit social interaction. Sci. Rep..

[46] Grossberg S (2013). Adaptive resonance theory. Scholarpedia.

